# Analysis of attitudes and practices toward gastroesophageal reflux disease among the general population of Pakistan

**DOI:** 10.2144/fsoa-2023-0144

**Published:** 2024-06-06

**Authors:** Humaira Jabbar, Tooba Noor, Muhammad A Obaid, Areej Shakil, Muhammad I Obaid, Syeda I Aaqil, Usama AA Memon, Zoha Mohsin

**Affiliations:** 1 Department of Medicine, Jinnah Sindh Medical University, Karachi, Pakistan; 2 Department of Family Medicine, Ziauddin University, Karachi, Pakistan; 3 Department of Medicine, Bahria University Medical & Dental College, Karachi, Pakistan

**Keywords:** attitude, gastro-esophageal reflux disease, GERD, knowledge, meta-analysis, practice, randomized controlled trials

## Abstract

**Aim:** Gastro-esophageal reflux disease (GERD) is a growing health concern. **Methods:** In this cross-sectional study, participants' knowledge, attitude and practice toward GERD were assessed using a questionnaire. **Results:** In our study of 411 participants, 92.5% knew about GERD. Correctly identified risk factors were smoking (62.3%), fatty food (84.2%), spicy food (91.2%) and meal timing (80.8%). Identified symptoms were burning sensation (92.2%) and regurgitation (81.0%). 43.6% of participants were unaware of GERD's complications. Only 46.2% would seek medical advice for feeling full after eating, but 85% would for severe symptoms. 88.7 and 86.8% of participants showed willingness to amend diet and lifestyle. **Conclusion:** General population has knowledge regarding GERD and its risk factors but poor attitude and practices toward the condition

## Plain language summary

**What is this summary about?:**

This summary presents the findings of a survey conducted to gauge people's understanding, attitudes and behaviors concerning gastroesophageal reflux disease (GERD). GERD is a condition involving the movement of stomach contents back into the esophagus or mouth, which can lead to the development of symptoms or complications. Common risk factors associated with the emergence of GERD symptoms, such as heartburn and the regurgitation of stomach contents into the oropharynx, include smoking, consumption of fatty and spicy foods and meal timing. While people are aware of these risk factors and the signs of GERD, many do not take proactive steps to seek medical assistance or make lifestyle changes. The goal of this study is to assess people's knowledge, attitudes and practices regarding GERD.

**What were the results & what do they mean?:**

In Pakistan, most people have good knowledge of GERD, but there's still a knowledge gap with important public health implications. Greater awareness and education are needed to improve GERD management and health outcomes for those affected.

**Who is this article for?:**

The authors of the original article developed this summary to help patients, caregivers, patient advocates, healthcare professionals, policymakers and insurance providers to understand the results of their study.

## Abstract (plain language summary)

Results showed that the majority of participants had knowledge of GERD risk factors, symptoms and treatments.

A considerable number of participants were unaware of the potential long-term complications of GERD, such as tooth decay and cancer.

Many participants indicated that they would seek medical attention only if they experienced severe symptoms, leading to delayed healthcare visits.

The study highlights the need for improved awareness and education about GERD and its potential consequences among the Pakistani population.

Health education programs should focus on the seriousness of GERD and the importance of early diagnosis and appropriate management.

By addressing knowledge gaps and promoting early intervention, the burden of GERD can be reduced, improving the quality of life for affected individuals.

Gastroesophageal Reflux Disease (GERD) is a frequently diagnosed disorder by healthcare practitioners over the globe [1]. It is a chronic condition characterized by troublesome symptoms and associated complications that occur when acidic stomach contents are refilled into the esophagus [2]. Its classic symptoms include heartburn alongside regurgitation and several less typical symptoms including dysphagia, chest pain that is not a sequel of any cardiac ailment, and a variety of ear, nose and throat complications [3]. Added to this, it has a severe negative impact on daily productivity and may have corollaries ranging from erosive esophagitis [4,5] ulcers, esophageal carcinoma and strictures [6,7].

Generally, GERD is common in North America and Europe where reported prevalence is 10–20% in the general adult population and 10–20% of the population have episodes of heartburn at least once a week Within North America, 20–30% of the US population experience primary symptoms of GERD per week [8] where it is considered a major healthcare problem [9]. However, epidemiological evidence has indicated a much lower, though increasing prevalence in Asia that remains at only 5% [10]. Among the Asian countries, India and Pakistan have a high incidence of GERD with the prevalence of 7.6–18.7% and 24–35%, respectively [11,12]. Additionally, the worldwide age-standardized rate (ASR) for esophageal carcinoma that may result as a complication of GERD is 7.7 per 100,000 individuals in Asia, with the highest ASR observed in Bangladesh and China, ranging from 12.5 to 12 per 100,000, while Pakistan reports a lower ASR of 4.1 per 100,000 individuals [13].

As stated earlier, the increasing prevalence of GERD in Asia is an alarming situation that needs immediate attention. Since the statistics are not updated for the last decade, our research aims to obtain the latest data and help us formulate better ways and methods to increase knowledge and promote a positive attitude toward GERD so that it can be diagnosed in its initial stage and prevent it from progressing into complicated scenarios. The aim of the study is to provide a descriptive analysis regarding the knowledge and attitude of the Pakistani population toward GERD, and highlight the reasons for delaying healthcare visits until the disease progresses to a severe level.

## Materials & methods

### Study setting & participants

The study was conducted in the general population of Karachi. Both male and female participants were chosen.

### Study design & sampling technique

It was a cross sectional study to assess knowledge and attitude about GERD among the Karachi population. Convenience sampling was used to recruit participants, by distributing both physical and online forms. Only the ones who agreed to take part in the study were included.

### Sample size

A minimum of 384 participants were chosen based on the sample size calculation formula

$$\text{n}=\frac{Z^{2}*p*(1-p)}{E^{2}}$$

Where n is necessary sample size, Z is Z-score; p is standard deviation; E is the margin of error.

$$\text{n}=\frac{1.96^{2}.*0.5*(1-0.5)}{0.05^{2}}=384.16$$

### Data collection & procedure

A pilot study on 30 subjects was done before interviewing the participants. The study depicted that the questions were well-understood by the participants; there was no confusion. The interview which was conducted, had 29 questions divided into six sections. The questionnaire was designed to study the knowledge and attitude of Pakistani people toward GERD, its symptoms and risk factors. To ensure participants' honesty, full anonymity was provided – their name, contact number and any personal details were not asked. Interviewer bias was reduced by training the interviewers. Miscommunication was prevented by appointing interviewers who were fluent in English/Urdu or other commonly spoken dialects. Informed/verbal consent was taken before the distribution of questionnaires. Participants were informed about the confidentiality of their responses.

### Inclusion & exclusion criteria

The subjects included in this study were those who had given informed/verbal consent and were healthy individuals between the ages 18–75 years.

The subjects excluded from the study were the ones who had a history of malignant disease, cardiovascular disease, renal failure, cirrhosis, rheumatic disease and pre-existing psychological disorder. Subjects taking steroids during the last 2 weeks and subjects below 18 years or above 75 years were also excluded from the study. Subjects who refused to give consent were excluded as well.

### Data collection tool

A structured questionnaire was used for the study. A section on demographics was included to collect data regarding age, gender, height, weight, area of residence, marital status, type of family system, extent of education, employment type and number of working hours. The rest of the questionnaire was divided into three major sections: knowledge of GERD, its risk factors and symptoms, attitude toward the disease, and the subject's practices regarding GERD including treatment modalities. The questionnaire mainly consisted of multiple-choice questions along with some checklist questions (Supplementary Appendix 1).

The first section of the questionnaire assessed the knowledge of participants regarding the risk factors of GERD (eating spicy food, eating fatty food, smoking, posture, tight belts, meal times, stress, pregnancy and obesity). Participants were asked which gender they felt was more at risk for getting GERD. Their knowledge of symptoms of GERD (burning sensation in chest, sensation of lump in throat, difficulty swallowing, chest pain and regurgitation of food/drink) was assessed.

In the second section of the questionnaire, the participants were assessed on their attitude toward GERD. They were asked how strongly they agreed with the fatality of GERD and whether it led to cancer and tooth decay or not. It was enquired if they had noticed any symptoms of GERD themselves and how did they know about GERD (through TV, educational programs, a doctor, a family member, social media or the internet). We asked the participants if they would see a doctor in the following cases or not: if they experienced pain/difficulty in swallowing, if they experienced frequent vomiting, if they felt full after eating, if they had chronic hoarseness, or any breathing problems. The participants were questioned if they would meet the doctor within 1 week, in 1–2 weeks or in more than 2 weeks. It was then assessed what factors restrained the participant from visiting a doctor (inability to take time from work or family, lack of transport, anxiety about seeing the doctor, or due to personal beliefs regarding healthcare).

In the third section of the questionnaire, the participants were assessed on their practice toward GERD. They were asked if they take any painkillers, such as omeprazole, pantoprazole, esomeprazole or antiacids like magnesium oxide etc., or change their position to relieve the symptoms. It was enquired how long they monitored their symptoms even after their treatment had started; was it till the symptoms resolved, or when the medications were over, or when the doctor said no more meds were required. The participants were also asked if they would change their smoking habits, eating habits, lifestyle and intake of late meals and fatty food to avoid GERD.

### Data analysis

Data analysis was conducted using Statistical Package for the Social Sciences (SPSS) version 25.0 by IBM. For categorical variables, e.g., gender, marital status, frequencies and percentages out of the total sample population were reported. For continuous variables e.g., age, weight and height, mean and standard deviations were reported.

## Results

### Demographics

**Figure 1.**
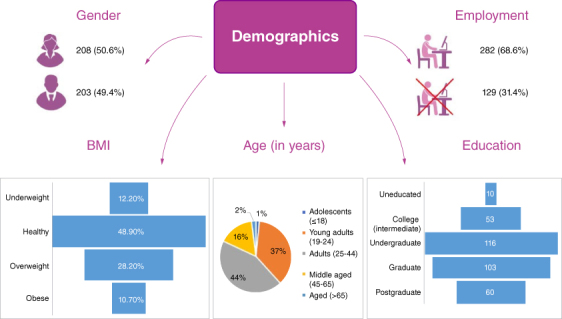
Demographics of the study.

Initially, our study included 411 participants, but the final sample size was smaller due to non-responsiveness, resulting in a sample size of 384. The participants had the mean age of 32.1 years and standard deviation of 12.8 years. The majority of our participants were adults (n = 179, 43.6%), followed by young adults (n = 153, 37.2%). The most frequently reported BMI class by our sample was healthy (n = 201, 48.9%) and overweight (n = 116, 28.2%). The majority of our participants (n = 380, 92.5%) had knowledge of GERD. The majority of our participants (n = 208, 50.6%) were females. The most common residential areas in our sample were Gulistan-e-Jauhar (n = 45, 10.9%), Gulshan-e-Iqbal (n = 39, 9.5%) and North Karachi (n = 35, 8.5%). Almost half of our participants were unmarried (n = 205, 49.9%). More than half of our participants lived in a joint family (n = 207, 50.4%). Our sample included participants who were postgraduates (n = 60, 14.6%); graduates (n = 103, 25.1%); and undergraduates (n = 116, 28.2%), followed by participants who had received education till intermediate (n = 53, 12.9%), matric (n = 48, 11.7%) madrassa (n = 21, 5.1%), and those who had no education (n = 10, 2.4%). The most frequently reported monthly income by our participants was 10,001 to 50,000 (n = 157, 38.2%) Pakistani Rupee per month. Unemployment was more frequently reported (n = 129, 31.4%), in comparison to any other employment type. The most common employment type was business (n = 72, 17.5%), followed by private work (n = 59, 14.4%). The most commonly reported working hours by our sample were between 4 to 8 h per day (n = 148, 36.0%) and more than 8 h per day (n = 146, 35.5%). These findings have been summarized in Figure 1 & Table 1.

**Table 1.** Demographics of the study population.

Factors		n	%
Age (years)	Adolescents (≤18)	6	1.5
	Young adults (19–24) Adults (25–44) Middle aged (45–65) Aged (>65)	153 179 65 8	37.2 43.6 15.8 1.9
Gender	Male Female	203 208	49.4 50.6
Marital status	Married Unmarried Divorced Widowed	193 205 8 5	47.0 49.9 1.9 1.2
BMI (kg/m^-2^)	Underweight (<18.5) Healthy (18.5–24.9) Overweight (25.0–29.9) Obese (≥30.0)	50 201 116 44	12.2 48.9 28.2 10.7
Monthly income PKR	None <10,000 10,001–50,000 50,001–100,000 >100,000	88 10 157 81 75	21.4 2.4 38.2 19.7 18.2
Working (h/day)	≤4 4–8 >8 None	11 148 146 106	2.7 36.0 35.5 25.8
Family system	Joint Nuclear	207 204	50.4 49.6
Education	Uneducated Madrassa Matric Intermediate Undergraduate Graduate Postgraduate	10 21 48 53 116 103 60	2.4 5.1 11.7 12.9 28.2 25.1 14.6
Employment	Banking Private Retired Services Unemployed Business Driver Education Engineering Government Healthcare Information technology Marketing	5 59 7 32 129 72 7 30 9 6 35 12 8	1.2 14.4 1.7 7.8 31.4 17.5 1.7 7.3 2.2 1.5 8.5 2.9 1.9
Knowledge about gastroesophageal reflux disease	Yes No	380 31	92.5 7.5

### Knowledge

**Figure 2.**
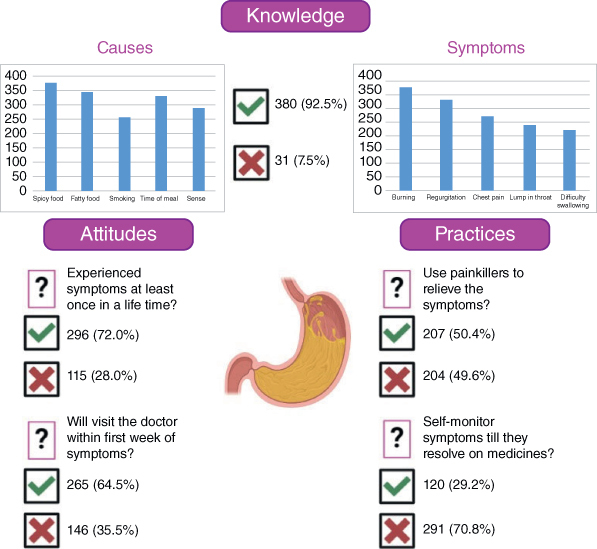
Summary of the results.

The majority of our sample reported that GERD occurred due to spicy food (n = 375, 91.2%), fatty food (n = 346, 84.2%), smoking (n = 256, 62.3%), time of meal (n = 332, 80.8%), stress (n = 287, 69.8%), obesity (n = 286, 69.6%) and pregnancy (n = 215, 52.3%). However, less than half of our sample reported GERD occurring as a result of posture (n = 192, 46.7%) and the use of tight belts (n = 160, 38.9%). Furthermore, more than half of the participants reported both genders (n = 236, 57.4%) at equal risk of GERD. The majority of our participants reported a burning sensation in the chest (n = 379, 92.2%) and regurgitation of food or drink (n = 333, 81.0%) as symptoms of GERD. Moreover, more than half of our participants reported chest pain (n = 275, 66.9%), sensation of a lump in the throat (n = 241, 58.6%), and difficulty swallowing (n = 224, 54.5%) as symptoms of GERD. The results are summarized in Figure 2.

### Attitude

More than half of our sample recognized GERD as a fatal disease (n = 245, 59.7%), with a high number of participants (n = 232, 56.4%) recognizing GERD as a factor for developing tooth decay or cancer. The majority of our participants (n = 296, 72.0%) had experienced symptoms of GERD at least once in their lifetime. The most common medium for acquiring awareness regarding the disease was thorough a doctor (n = 127, 30.9%) or a family member (n = 124, 30.2%), followed by educational programs (n = 53, 12.9). The majority of our participants reported that they would consult a healthcare professional if they had episodes of frequent vomiting (n = 351, 85.4%) or chronic hoarseness/breathlessness (n = 346, 84.2%). More than half of the participants also reported visiting a doctor if they experienced pain or difficulty in swallowing (n = 300, 73.0%). In contrast, less than half of our participants would visit a healthcare professional in the case of feeling full after eating (n = 190, 46.2%). Most participants would visit a doctor within the first week (n = 265, 64.5%). However, the most frequently reported reason for preventing seeing a healthcare professional by our participants was the inability to take time from work (n = 239, 58.2%), followed by personal beliefs on healthcare (n = 199, 48.4%), anxiety before visiting a doctor (n = 175, 42.6%), inability to take time from family (n = 156, 38.0%), whereas, the least reported reason was lack of available transport (n = 141, 34.3%). The results are summarized in Figure 2.

### Practice

Almost half of our participants reported using painkillers to relieve the symptoms (n = 207, 50.4%), whereas more participants reported changing positions to relieve symptoms (n = 287, 69.8%). Most of our participants reported monitoring symptoms till the symptoms (n = 120, 29.2%) resolve after treatment has started. The majority of our participants reported that they would change eating habits (n = 322, 88.7%), avoid late meals and high-fat diets (n = 332, 89.2%) and modify their lifestyle (n = 317, 86.8%), and would quit smoking (n = 99, 76.1%) to relive their symptoms. The results are summarized in Figure 2.

## Discussion

As evidenced by the statistics, GERD has shown a substantial increase over recent decades across the globe, hence, there is a dire need for early recognition of this disease which is becoming increasingly difficult because of the erroneous public impression of GERD symptoms being negligible with no long-term health consequences. The patients do not perceive their condition seriously and wrongly assume that self-medication with over-the-counter (OTC) medicines is enough to manage their symptoms [14]. In some cases, particular foods, imbalance in eating with greater intake of fats, eating too late, and obesity trigger GERD while in other cases, stress, smoking, and lying down can provoke GERD symptoms [15]. The management of GERD in the early stage depends on lifestyle modifications such as cessation of smoking, alcohol and tobacco together with decreased consumption of spicy citrus food and carbonated or caffeinated beverages. However, in the progressive stage, pharmacological measures like using proton-pump inhibitors that are easily available over the counter drugs are a requirement for the successful management of the disease [16,17]. As GERD is a potentially crippling disease, a significant number of patients suffering from GERD have impaired quality of life as much so that it is ranked worse than that of patients with hypertension, angina, mild congestive heart failure or diabetes (Types I and II combined) [18]. GERD affects several domains of life; regurgitation causes irritation to the esophagus resulting in severe pain and discomfort and hence interferes with feeding and sleeping. Subsequently, work productivity is negatively affected among patients with GERD symptoms [19]. In addition to this, affected patients of GERD also experience emotional distress, negative impact on sexual relations and limitation of social activities [20].

To the best of our knowledge, this is the first national study in Pakistan to report the knowledge, practice and attitude toward GERD among the Pakistani population. Our study sought to gain a deeper understanding of the general public's knowledge, attitude and practice concerning GERD in Pakistan. Another purpose was to evaluate why people hesitate in consulting their symptoms to a specialist until the disease has worsened. The actual benefits of this study are raising awareness of GERD and its complications among the people of Pakistan and comparing various groups in the target population, such as different genders, educational levels, monthly income and others, to see the difference between their awareness of this disease and its complications. The practical benefits for society also include directing local health education programs' efforts on actual GERD needs and attempting to concentrate on particular groups in a population if there is a discrepancy in the level of knowledge. Patients will also be made more aware of essential behavioral changes, such as diet, medication use and seeking early professional care, as a result of an increased understanding of the disorder.

Based on the results of our questionnaire, 92.5% of the participants had knowledge of GERD, this high percentage of knowledge may be a result of an increase in the prevalence of symptoms of GERD over the past few decades which was reported to be 22.2–24.0% according to several hospital-based studies in Pakistan and is still on the rise with recent changes in lifestyle [21]. If this pattern persists, it may contribute to the fast-rising prevalence of more severe GERD consequences, which would have an impact on patients' quality of life as well as the financial burden on healthcare systems. More than half of our participants lived in a joint family, and nearly half of our participants were unmarried. The most often stated working h, if they were employed, were between 4 and 8 h per day but unemployment was more commonly reported in the participants.

The majority of our participants correctly reported smoking, oily and spicy food, and the timing of meals as considerable risk factors for GERD. This corroborates with previous findings, where knowledge about GERD risk factors is consistent according to the univariate analysis that demonstrated statistical significance and connection (p < 0.05) with symptoms of GERD and smoking, a high BMI (>25 kg/m^2^), fast food, and sleeping within one hour after dinner [22]. Smoking relaxes ring muscles in the lower esophageal sphincter increasing the chances of acid regurgitation hence making it a significant factor, which is consistent with the findings of numerous earlier research [23]. Moreover, research in an Indian population, which shares several cultural traits with Pakistan, found a link between GERD symptoms and eating fried food [24]. In addition to this, in subjects from Pakistan, there was a correlation between eating spicy food and worsening of GERD symptoms [25]. These foods' results are facilitated by lower esophageal sphincter relaxation.

Moreover, the majority of the respondents recognized a burning sensation in the chest and regurgitation as major symptoms of GERD. This may be explained by the fact that heartburn and regurgitation are the two most typical symptoms, experienced by almost 15% of people in the general population on a weekly basis [26]. Heartburn can be differentiated from an intense scorching pain in the retrosternal area that lasts for several minutes. Due to reflux, patients may experience chest pain that resembles angina [27].

Our analysis demonstrated that 57.4% of the sample reported an equal risk of GERD for both genders, which is in line with another observation from our study where associations between GERD symptoms and gender were notably similar between male and female participants (68.9% and 75%, respectively). This finding is consistent with another study conducted in Karachi, Pakistan, and in Arar, Saudi Arabia [28,29]. Contrastingly, descriptive analysis from South India opposed this evidence, concluding a significant association of male gender and GERD [30], so there have been inconsistent findings, and further research is necessary to determine whether gender is a related risk factor for GERD. Moreover, 46.7% of the responders were aware that posture could be one of the risk factors of GERD, lying down after meals in the supine position is thought to lessen lower esophageal sphincter pressure, which explains this relationship [31].

Our findings highlight that the general public of Pakistan had a strong comprehension of the risk factors, such as particular meals and smoking, and was knowledgeable of the typical GERD symptoms. However, it is unlikely that patients will apply this knowledge until they are better educated about the serious complications and morbidity associated with GERD, as a large number of people (43.6%) in this study were unaware that this disease can lead to serious complications such as tooth decay or cancer in the long run. Due to their ignorance of GERD, they may wait until their symptoms are severe before seeking therapy. Information regarding GERD must be distributed at medical appointments and in the community. Asking about both the typical and related symptoms will help patients become more aware of any symptoms they might mistake for indigestion or other minor illnesses. Moreover, the identification of risk factors and triggers, GERD symptoms, dietary and lifestyle changes to lessen the severity and frequency of symptoms, appropriate over-the-counter drugs, and instructions for seeking medical attention should all be covered in education.

According to studies from Pakistan, the majority of GERD patients do not seek medical help and those who do typically exhibit symptoms, both many and severe, than those who do not [22]. This is in accordance with the results of our study, as only a small percentage of respondents indicated that they would seek medical advice if they experienced symptoms like feeling full after eating, but 85% indicated that they would do so if they experienced severe symptoms like frequent vomiting or chronic hoarseness. The symptomatic alleviation that comes from self-medication with antacids and painkillers may help to partially explain why only a few individuals in our society seek medical attention. The majority of our participants were amenable to dietary and lifestyle changes, such as skipping spicy or late-night meals to avoid GERD symptoms. Because dietary and lifestyle changes are typically the first line of treatment for GERD patients, this raises the possibility of reducing the number of GERD patients to a very low level.

Our findings have certain limitations. Certain neighborhoods were purposefully chosen in order to reach our desired response rate, potentially leading to selection bias. Moreover, the influence of translators who were all medical professionals and aware of survey research could influence how respondents answered the questionnaire. Additionally, any use of ambiguous terminology by interviewers could have led to participant misinterpretation. Moreover, a third of participants had a medical background and the majority of participants had higher education levels. Therefore, the inclusion of such respondents and the lack of a stratified analysis limits the ability to generalize our findings to uneducated individuals in the general public of Pakistan. Last, since our observations were limited to participants in Karachi, Pakistan. Since Karachi represents an urban metropolis, therefore, our results should be cautiously extrapolated to the entire population of Pakistan, including the rural population which may have entirely different demographics compared with individuals in an urban center of the country. Moreover, the sample used in our study consisted only of volunteers who agreed to take part in the survey, therefore it is not genuinely representative of the entire Pakistani community. In extension to this, it is possible that participants who had symptoms were more likely to give consent for the survey.

Albeit certain limitations, our findings have several clinical implications. Our findings help clinical decision-makers target academic interventional approaches to improve knowledge and awareness regarding GERD. By providing a descriptive analysis of existing knowledge and gaps regarding the perception of GERD, our findings emphasize the development of individualized treatment plans and adherence to existing treatments to curb the burden of GERD in a developing nation. Our analysis also highlights the limitations of existing data regarding GERD among various cultural and ethnically diverse groups that exist in Pakistan and highlights future avenues of research for investigators to evaluate cultural and ethnic factors that impact perception toward GERD. Additionally, our study provides a summary of existing barriers and challenges in GERD management, in terms of patient inertia toward clinical diagnosis. This can help clinicians understand the aspects to implement evidenced educational approaches to improve the management of GERD among the general population of Pakistan. In the future, a rigorous longitudinal comparative analysis is warranted to assess the efficiency of GERD interventions and compare and contrast the results with various Western and European populations to evaluate the impact of such interventions and factors that may hinder or improve the propagation of knowledge among the general population. Lastly, studies are warranted with the aim to assess the perspective of healthcare practitioners regarding challenges in the management of GERD in the Pakistani population.

## Conclusion

In conclusion, the majority of the general population of Pakistan had profound knowledge regarding the disease. However, a potential gap in knowledge has important implications for public health and highlights the need for greater education and awareness about this condition. With the right support and resources, it is possible to reduce the impact of GERD and improve the health outcomes of those affected by this condition.

## Supplementary Material

Supplementary Appendix 1

## Supplemental material

To view the supplementary data that accompany this paper please visit the journal website at: www.future-science.com/doi/suppl/10.2144/fsoa-2023-0144
