# Supplementary material for: Analysis of attitudes and practices toward gastroesophageal reflux disease among the general population of Pakistan
Source: Future Sci OA. 2024 Jun 6;10(1):2359843. doi: 10.2144/fsoa-2023-0144 (PMC11160512; doi:10.2144/fsoa-2023-0144)
Supplement: Supplementary Appendix 1 [file IFSO_A_2359843_SM0001.docx]

GERD Questionnaire

* Indicates required question

1. Do you give consent to participate in this research? *

*Mark only one oval.*

Yes No

Demographics

1. 1. Do you know about gastroesophageal reflux disease/Acidity/Taizabiyat *

*Mark only one oval.*

Yes No

# 2. Age *

1. 3. Gender *

## Mark only one oval.

Male Female

1. 4. Height *
2. 5. Weight *
3. 6. Area of residence *
4. 7. Marital status *

## Mark only one oval.

Married Unmarried Divorced Widowed

# 8. Family system *

## Mark only one oval.

Joint Nuclear

# 9. Education *

## Mark only one oval.

Matric Intermediate Undergraduate Postgraduate Madrassa Graduate

Untitled Section

1. 10. Monthly household income *
2. 11. Employment type *
3. 12. Working hours *

Knowledge of GERD

# 13. Do you think the following are risk factors of GERD/Acidity/taizabiyat? *

*Check all that apply.*

Eating spicy food

Eating fatty food

Smoking Posture

Tight belts

Meal times

Stress Pregnancy Obesity

Yes No

# 14. Which gender is at more risk for GERD/acidity/taizabiyat? *

## Mark only one oval.

Male Female Both

# 15. Do you think the following are the symptoms of GERD/acidity/taizabiyat? *

*Check all that apply.*

Yes No

Burning sensation in chest

Sensation of a lump in your throat

Difficulty swallowing

Chest pain

Regurgitation of food or drink

Attitude

# 16. Do you think GERD/acidity/taizabiyat is a serious and fatal disease? *

## Mark only one oval.

Strongly agree Agree

Neutral Disagree

Strongly Disagree

# 17. Do you think GERD/acidity/taizabiyat can lead to cancer and tooth decay? *

## Mark only one oval.

Yes No

# 18. Have you ever noticed any signs or symptoms of GERD/acidity/taizabiyat? *

## Mark only one oval.

Yes No

# 19. Where did you learn most about GERD/acidity/taizabiyat? *

## Mark only one oval.

TV

Educational programs Doctor

Family member Social media Internet

# 20. If you had the following experiences, would you see a doctor? *

*Check all that apply.*

Yes No

Pain or difficulty swallowing

Frequent vomiting

Feeling full after eating

Chronic hoarseness

⁄ breathing problems

# 21. When will you go to see the doctor if you notice the symptoms? *

## Mark only one oval.

Within 1 week

1 to 2 weeks

More than 2 weeks

# 22. Issues that prevent you from seeing a doctor if you had heartburn symptoms *

*Check all that apply.*

Yes No

Inability to take time from family

Inability to take time from work

Does not have transportation

Anxiety about seeing doctor

Personal beliefs on healthcare

Practice

# 23. Do you take any painkillers to relieve symptoms? *

## Mark only one oval.

Yes No

# 24. Do you change positions to relieve symptoms? *

## Mark only one oval.

Yes No

# 25. How long do you monitor your symptoms even after the treatment has started? *

## Mark only one oval.

Till the symptoms resolve Till the medications are over

Till the doctor says no more medication is required

# 26. Will you stop smoking to avoid GERD/acidity/taizabiyat? *

## Mark only one oval.

Yes No

I don't smoke

# 27. Will you change your eating habits to avoid GERD/acidity/taizabiyat? *

## Mark only one oval.

Yes No

Does not apply

# 28. Will you change your lifestyle to avoid GERD/acidity/taizabiyat? *

## Mark only one oval.

Yes No

Does not apply

# 29. Will you avoid late meals and high fat diets? *

## Mark only one oval.

Yes No

Does not apply
